# When the Ideal Meets the Feasible: Constructing a Protocol for Developmental Assessment at Early School-Age

**DOI:** 10.3389/fped.2018.00256

**Published:** 2018-09-24

**Authors:** Adel Farhi, Saralee Glasser, Shay Frank, Galit Hirsh-Yechezkel, Louise Brinton, Bert Scoccia, Rafael Ron-El, Liat Lerner-Geva, Lidia V. Gabis

**Affiliations:** ^1^Women & Children's Health Research Unit, Gertner Institute for Epidemiology & Health Policy Research Ltd., Sheba Medical Center, Ramat Gan, Israel; ^2^Weinberg Child Development Center, Safra Children's Hospital, Sheba Medical Center, Ramat Gan, Israel; ^3^Division of Cancer Epidemiology & Genetics, National Cancer Institute, Rockville, MD, United States; ^4^Division of Reproductive Endocrinology & Infertility, University of Illinois College of Medicine, Chicago, IL, United States; ^5^Infertility & IVF Unit, Department of Obstetrics & Gynecology, Assaf Harofe Medical Center, Ramat Gan, Israel; ^6^Sackler Faculty of Medicine, Tel Aviv University, Tel Aviv, Israel

**Keywords:** assessment, child development, cognitive, health, parents, telephone interview, learning disorder, ADHD

## Abstract

**Objective:** To describe development of a methodology for an outcome study of children born following *in-vitro* fertilization or spontaneously-conceived, as a model for defining normal and below-normal development of school-age children for research purposes.

**Study Design:** The main issues addressed were defining the major health and developmental domains to be investigated, selection of age-appropriate validated instruments, considering time constraints to maximize compliance, and budgetary limitations. The final protocol included a half-hour structured telephone interview with mothers of all 759 children and a 2-h developmental assessment of 294 of them. Each of the instruments and recruiting methods are described in terms of the abovementioned considerations.

**Results:** Almost all of the mothers who agreed to be interviewed completed it within the half-hour allotted; however only about half of those who agreed to bring the child for the developmental assessment actually did so. The entire examination battery, assessing cognitive ability, executive functions, attention, and learning skills, was completed by almost all 294 children. There was a significant degree of agreement between the maternal report of the child's reading, writing and arithmetic skills and the in-person examination, as well as regarding the child's weight and height measurements.

**Conclusion:** The findings lend support for a low-budget study, relying on telephone interviews. However, limitations such as the validity of maternal report and recall bias must be taken into consideration.

## Introduction

Studies investigating developmental outcomes of pre- and perinatal interventions frequently require assessments during the early school years. By this age, major developmental diagnoses have usually been established, and the diagnostic tools for autism spectrum disorders (ASD), cerebral palsy and intellectual impairment are well-defined. Selecting tools for examining major developmental diagnoses are areas of general consensus, and protocols are relatively straight forward. However, for research purposes a much more difficult task is identifying areas of below-clinical-threshold developmental concerns, minor disabilities, or defining the “normal school-age child.” In this age group, specific minor diagnoses, such as learning disorders and attention-deficit hyperactive disorders (ADHD) just begin to emerge and continue to change, leading to their being a “moving target.” Thus researchers in the field of child development are often faced with complex decisions with respect to constructing relevant and feasible research protocols. In the current report we share our experience, i.e., the deliberations and considerations, in developing the methodology for an outcome study of children 7–9 years of age who were born following *in-vitro* fertilization (IVF) treatments or spontaneous-conception (SC), as a model for defining normal and below-normal development of school-age children for research purposes.

Since the first IVF birth, the rate of these treatments has been steadily increasing worldwide (1). In 2011, 4.1% of all live births in Israel were conceived with IVF (2). However, despite the wide use of IVF, there is still concern regarding its safety. An Israeli study followed a cohort of IVF and SC pregnancies (3). That study cohort served as the basis from which this follow-up study was conducted. The challenge was to define a comprehensive assessment that would provide accurate outcome information on this large cohort. Planning of the protocol had to take into account the content of the health and developmental domains to be covered, age-appropriateness and validity of the instruments, and constraints such as time and budget.

## Methods

### Participants

The study cohort included children conceived either following IVF treatments or spontaneously, whose mothers participated in the original study, having been recruited from June, 2006 through December, 2008. This prospective cohort was identified in early pregnancy or randomly selected from delivery room records (3). Telephone interviews were conducted 6 weeks following delivery regarding the course of pregnancy and birth outcomes. All women who agreed at that time to be contacted in the future, constituted the basis for the present study population.

### Considerations for selection of study instruments

#### Content

To cover as broad a spectrum of the children's health and development as possible, emphasizing minor deficits, the study aimed to gather information on the following domains: demographic data; child's medical history, including perinatal data; developmental information, including diagnoses, need for services and interventions; education; gross and fine motor development and coordination; ASD traits and symptoms; emotional or behavioral problems, including ADHD; sensory processing disorder; cognitive development (intelligence, neuropsychological, achievement), including executive functions, language skills, reading, writing and arithmetic; motor planning and grapho-motor integration; anthropomorphic measurements—weight, height, and head circumference.

#### Age-appropriateness

Since the children ranged in age from 7 to 9 years at the time of this study, it was necessary to select instruments that were appropriate for these ages. An advantage could be if the instrument was also appropriate for later ages, in the event that a further follow-up study might be planned.

#### Validity and language

Many screening questionnaires have been developed and validated in English. Since the present study would include only Hebrew speakers, preference was given to instruments that had been translated into Hebrew, and validated or used in Israel. Having Israeli norms for the instrument was considered an advantage for selection, but not necessary for the purposes of the current study, since the outcomes were in terms of comparisons between the two study groups, rather than relative to the general Israeli population.

#### Time constraints and compliance

In the hope of gathering all information possible about the maximum number of children, it was decided that whatever could be accessed from the mothers would be included in the telephone interview protocol. Based on previous experience, the optimal length of such an interview would be about 30 min; thus the number of items had to be taken into consideration, with preference given to briefer, but valid, instruments. The children's examination, conducted by developmental psychologists at a Child Developmental Center, was to be limited to one session, since it was doubtful that parents would bring their child more than once. Time-wise, the psychologists recommended that the session be no longer than 2 h.

#### Budget

As with any research, there were budgetary limitations, and in this case the cost for skilled personnel was a priority. There were two phases to the study: the telephone interview with mothers of all children and an examination of a sample of the cohort. Telephone interviewers had to be experienced in encouraging participation and sustaining compliance throughout the interview. They also had to be familiar with the medical and developmental vocabulary and able to relay the structured questionnaires in an optimal manner. The more in-depth neurodevelopmental assessments required skilled and trained psychologists. Although the examiners were professional psychologists, in order to ensure the accuracy and reliability of the assessments, a senior psychologist was also employed to supervise conducting and scoring of the instruments. In addition to personnel, many research instruments are copyrighted and their use is conditional on payment. On the other hand, some instruments are either offered at reduced cost for research purposes, or permission is given free of cost. The latter was obviously an advantage, although in no case was it the determining factor in the selection.

### Study instruments

The telephone interview included the following:

- **Demographic data update**. In addition to data gathered in the original study, updates included six items regarding family status, parents' level of education, religious affiliation, number of children in the family, and index child's order.
- **Children's medical history**. Birth data was drawn from the original study and updated with 15 items regarding vision and hearing disability, chronic illnesses, medications, hospitalizations, para-medical rehabilitative interventions (occupational, physical, and speech therapy), and diagnoses of ADHD and ASD, as well as current weight and height.
- **Children's educational achievement**. Six items regarding past and current educational framework (regular, special education, aides); diagnosed learning disorder; school achievement relative to peers (reading, writing, arithmetic).
- **Developmental Coordination Disorder Questionnaire** (DCDQ) (4). The DCDQ assesses motor performance (gross and fine) and coordination in daily life activities of children aged 5–15 years. It is a brief (15-item) questionnaire requesting that mothers compare their children's performance to that of same-age peers, and rate items on a 5-point Likert scale. The authors provide age-scaled cut-off scores indicating either “probably not DCD,” “suspect DCD,” or “indication of DCD." A total score is calculated, as well as scores on the three factor scales: control movement, fine motor and coordination. The Hebrew translation of the DCDQ has been validated in Israel (5). In the present study the standardized internal consistency of the DCDQ was α = 0.87. According to the Alberta Children's Hospital, Calgary, Alberta, Canada, the questionnaire and manual are available for use free-of-charge conditional on its being used in its entirety, with appropriate references.
- **Autism Spectrum Screening Questionnaire** (ASSQ) (6). Since there are conflicting data regarding heightened risk of ASD among children born following IVF, it was important to learn whether those who had not been diagnosed, nevertheless displayed ASD symptoms. The ASSQ is a 27-item parent report of such symptoms in children and adolescents with normal intelligence or mild mental retardation. Parents rate the children's behavior on a 3-point Likert scale indicating normality, some abnormality, and definite abnormality. Items relate to social interaction, communication problems, restricted and repetitive behavior, motor clumsiness and other associated symptoms (including motor and vocal tics). According to the authors a cutoff score of 13 yields a true-positive rate of 91%, and a cutoff score of 19 yields a true-positive rate of 62%. The ASSQ has been translated into Hebrew for an Israeli population by Shtaierman et al. (7). In the present study the standardized internal consistency of the ASSQ was α = 0.83. The instrument is available on the internet and its use is free-of-charge.
- **Child Symptom Inventory** (CSI-4) (8, 9). In order to obtain a broad picture of the children's emotional problems, the CSI-4 was selected. The 61-item CSI−4 is a DSM–IV-referenced rating scale used to screen for various childhood disorders. The parent assesses behavioral, affective and cognitive symptoms of several psychiatric disorders in children 5–12 years old. Several subscales of the CSI-4 parent version were used in this study: ADHD inattentive, hyperactive-impulsive, and combined disorder, oppositional defiant disorder, general anxiety disorder, specific phobia, obsessive-compulsive disorder, post-traumatic stress disorder, motor and vocal tics, major depressive disorder, dysthymic disorder, separation anxiety, and elimination disorder. Items are scored on a 4-point Likert scale. The current study scored the items according to “symptom count,” for which item scores are dichotomized (never/sometimes = 0; often/very often = 1). The CSI-4 has been validated (10) and was translated into Hebrew for use in Israeli research (11). There was no cost for use of the questionnaire, as the Hebrew version of the CSI-4 was used with permission of the authors.
- **Short Sensory Profile** (SSP) (12). The SSP was selected to assess sensory integration on various modalities as expressed in functional performance of activities in daily life. This 38-item parent questionnaire is a shorter version of the Sensory Profile (13) with seven subtests, including: tactile sensitivity, movement sensitivity, taste/smell sensitivity, under-responsive/sensation-seeking, auditory filtering, low energy/weak, and visual/auditory sensitivity. A total score is also calculated, which provides an indication of the children's general sensory processing ability. Items are scored on a 5-point Likert scale, with total and subscale scores reflecting either “typical performance,” “probable difference,” or “definite difference” compared to the normative population. The SSP has been translated into Hebrew and validated on Israeli children (14–16). The SSP has been used in a follow-up study of 739 children born at very low birthweight, and it clearly distinguished the study group from the normed population (17), thus it was hoped that it might be sensitive to more subtle differences than would the other instruments. In the present study the standardized internal consistency of the SSP was α = 0.90. Pearson Clinical Assessment Inc., which holds the rights to the SSP, granted permission to use the Hebrew translation and permission to administer the SSP was purchased under Pearson's Research License Agreement (05475-G), which afforded a discount when used for research purposes.

The in-depth neurodevelopmental assessments required skilled and trained psychologists, rather than undergraduate personnel. Depending on these professionals was considered crucial for several reasons: (a) since this was the first time using this specific battery of tests, it was expected that the psychologist would provide additional input if there were neurodevelopmental concerns beyond those picked up by the specific selected tests; (b) additional adjustments to the battery were planned according to the input from these examiners, so their subjective observations with regard to the children's performance were considered important; (c) since multiple tasks were to be completed within the 2 h allotted, the examiner had to be skilled enough to plan and engage the children in performing all of the tasks in one session; (d) the fact that experienced psychologists conducted the examinations improved parents' compliance and confidence in the assessment.

The battery of tests selected was chosen to cover all developmental areas that might be of concern at school age, with emphasis on diagnoses that are more difficult to assess at this age. The domains included were those generally used in standard neuropsychological assessments and considered essential for gaining a broad picture of the children's functioning: intelligence, language, attention, executive functions and grapho-motor integration. Didactic functions were added to identify children at risk for learning disorders. Additional considerations, as noted above, included appropriateness for this age group and brevity of the tool, while avoiding overlap between tools assessing different areas. Due to time constraints, for some tests only specific subtests were used. Although it was necessary to adapt scheduling of the assessments to the parents' convenience, efforts were made not to schedule for late afternoon hours when the children might be particularly tired. The examination included:

- **Kaufman Brief Intelligence Test** (K-BIT-2) (18). The K-BIT-2 is a clinical instrument for assessing the cognitive development of children aged 3–18. Although there are no Israeli norms, its brevity and comprehensive scope were considered an advantage. Subtests include two tests of verbal intelligence (receptive vocabulary and general information) and riddles (verbal comprehension, reasoning and vocabulary knowledge), and one test of non-verbal intelligence, matrices (solving new problems, perceiving relations and completing visual analogies not requiring oral responses, vocabulary or language skill). Some items on the KBIT-2 verbal tasks contribute to assessing acquired lexical knowledge. Verbal IQ, Nonverbal IQ, and Composite IQ are calculated according to age-based standard scores (19).
- **Kaufman Assessment Battery for Children** (K-ABC) (20). The arithmetic subtest of the K-ABC was added to the study protocol to assess didactic function and risk for learning disorder in this area. Age-based standard scores are calculated. Whereas the K-ABC has the advantage of Israeli norms, administration of the entire test was too long to meet the time limitations of the current study.
- **Test of Everyday Attention for Children** (TEA-Ch) (21). The TEA-Ch was employed to assess various components of attention and executive functioning. Two of the nine subtests were employed: Creature Counting, which involves attentional control and cognitive flexibility (switching), and Score!, which requires sustained attention. These tests have been found to distinguish between children with and without attention deficits. Age- and gender-based scores are calculated.
- **Beery-Buktenica Developmental Test of Visual-Motor Integration** (VMI) (22). The VMI was used to assess the grapho-motor integration ability. The children are asked to copy geometric forms arranged in a developmental sequence. The VMI is useful in identification of children at risk for developing writing difficulties in school. Age-based scores are calculated.
- **VMI Supplemental Test for Visual Perception** (22). This test assesses perceptual matching with limited motor task requirements. Participants are asked to match a given geometric form with one of several other similar forms, organized from simple to complex. This test was included since it reflects an important cognitive element of visual perception. Age-based scores are calculated.
- **Rey-Osterrieth Complex Figure Test** (ROCFT) (23, 24). The ROCFT is a neuropsychological assessment to evaluate various cognitive abilities, including attention and executive functions, with an emphasis on planning and organization, visuospatial perception, and motor coordination. Further, poor performance on this task has been noted among children with attention deficit or learning disorders, thus it has been found useful in identifying children at risk for these neuro-developmental difficulties. The children are asked to copy a complex form, organizing the figure into meaningful perceptual units. The approach to the activity, accuracy of the copy, and placement of elements of the figure are taken into account. There are various scoring methods for the ROCFT, and for this study the classical method developed by Osterrieth (25) was used, for which age-based scores are calculated. Due to the time constraint, the memory portion was not included.
- **Aleph-ad-Taf** (26, 27). This is the only instrument for assessing Hebrew reading and writing, and language elements related to acquisition of these skills. It is normed for children from the middle of second grade through sixth grade. The battery included three subtests: reading (single words and a paragraph) and writing (dictation). Both accuracy and speed were recorded. Grade-based scores are calculated.
- **Tavor Picture Naming Expressive Vocabulary Test** (28). The Tavor is the only Hebrew test for assessing expressive vocabulary and naming ability, which are central for language development. Children are shown a series of complex scenes and asked to name various objects or activities in each. Age-based scores are calculated for children up to eight years of age only.
- **Growth measurements**. Weight, height and head circumference were measured by the psychologist, with the parent's assistance if preferable to the child.

### Procedure

Recruitment was conducted when the children were ~7–9 years of age. Introductory letters were sent to all mothers in the original cohort who had agreed to be contacted in the future. The letter explained that they would be offered the opportunity to participate in a follow-up study. They were then contacted by telephone and those who agreed were interviewed. A pilot study was conducted to test the length and clarity of the questionnaire, and interviewer-training was conducted. Although minor modifications were made, it was not necessary to remove any of the main domains or to change any of the structured questionnaires. Interviewers were blind with regard to study group assignment.

At the conclusion of the telephone interview, mothers were asked if they would be willing to bring their child for developmental assessment at the Sheba Child Development Center. Those who consented were subsequently contacted to schedule the visit. Specific training for the study protocol tests was conducted. Scoring was done by the examining psychologists, who were blind with regard to study group assignment. Following the examinations, letters were sent to the parents summarizing the results of each test and including recommendations for further consultations, as determined by the examining psychologist and the Director of the Child Development Center.

### Statistical analysis

The internal consistency of the instruments used in this study was assessed as per the Chronbach α score (0–100%). Nominal variables were analyzed by Chi-square tests and ordinal or numeric variables were analyzed by Spearman or Pearson correlation coefficients. Analyses were conducted using SAS Version 9.4 (SAS Institute, Inc., Cary NC). A *P* < 0.05 was considered significant.

### Ethics

The research protocol and questionnaires were approved by the IRB Helsinki Committee of Sheba Medical Center (#3657).

## Results

**Figure 1:**
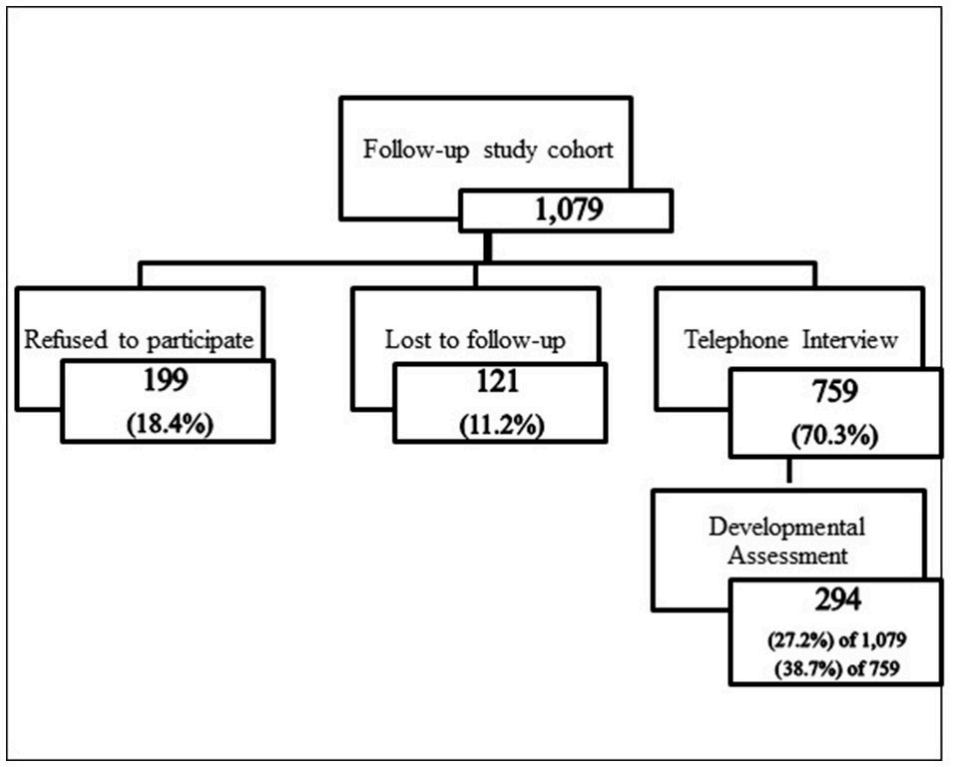
Participation in follow-up study.

After having been requested to participate in the follow-up study and respond to the half-hour telephone interview, the mothers of 759 children agreed (Figure 1). Most of the non-participants simply refused (*n* = 199), whereas many others (*n* = 121) could not be contacted due to change in residence, phone number, etc. Three mothers and one child were deceased. Almost all of the participants (97.2%) completed the interview within the half-hour allotted, although a few split the interview into two separate sessions. Those who were interviewed were more likely to have above-high school education than those who were not interviewed (69.3 vs. 56.7%, respectively; *P* < 0.0001) and less likely to have smoked during pregnancy (6.9 vs. 10.3%, respectively; *P* = 0.05). There were no significant differences between these two groups with respect to medical or obstetric history, or infant characteristics (prematurity, low birthweight, or congenital malformations).

Five-hundred and sixty-eight interviewees (74.8%) expressed willingness to bring their children for the developmental assessment. Ultimately, however, when contacted to make the appointment only 294 of them (51.8%) agreed to bring their children for examination, thus only 38.7% of the participants were actually assessed. Most of the remainder declined outright (*n* = 343), whereas the others either could not find a convenient time, made the appointment but did not arrive, or agreed on the condition that the assessment be done in the home (which was not feasible in the context of this study). Comparison of those who were unwilling to bring their children for the exam with those who agreed but did not actually come and those who actually brought their children for the exam indicated no differences with respect to the mothers' educational level, the children's age at the time of interview, or diagnosed ADHD or learning disorders. However, significantly more mothers who thought that their children's writing skill was worse than that of their peers brought their children to the exam (*P* = 0.02).

The battery of tests in the study protocol was completed within 2 h by the vast majority of children who came for the assessment; completion rates ranged from 100% for the KBIT-2 and the VMI-Copy, to 92.5% for the Tea-Ch Creature Counting (accuracy). For children with no difficulties 90 min was often sufficient; however, some children required up to two-and- a-half hours: those with attention difficulties and those who were more introverted or anxious, or conversely those with high levels of achievement whose responses continued to advanced levels. This time span also included some discussion with the parents for explanation and signing of informed consent and preliminary feedback at the end of the assessment.

An interesting aspect of the study design was the use of maternal report as well as actual examination of the children. Of interest was that the degree of agreement between the mothers' report of their children's reading, writing and arithmetic performance in comparison with classroom peers was significantly associated with the scores on the developmental assessment tests with some of the in-person assessments (Table 1). In addition, there was general agreement between the mother's assessment and the psychologist's determination of whether further consultation was required. In other words, mothers who assessed their children's school performance as better than that of their peers were more likely to receive a recommendation of 'no need' for consultation, and those who considered their children's performance worse than that of the peers were more likely to receive a recommendation for consultation (*P* = 0.0001 for reading and writing, and *P* = 0.08 for arithmetic). Further, the mothers' reports of diagnosed or suspected learning disorder were significantly associated with their children's performance on the TEA-Ch Creature Counting and Score! Tests (*P* = 0.0003 and *P* = 0.04, respectively), and their report of diagnosed or suspected ADHD was significantly associated with the TEA-Ch Creature Counting Test (*P* = 0.0002) and of borderline significance with the TEA-Ch Score! Tests (*P* = 0.10). The mothers' reports of their children's weight and height as measured in the past 6 months were also significantly related to the actual measurement, with correlation coefficients of 0.88 and 0.77, respectively (*P* < 0.0001 for both).

**Table 1:** Association between mother's report of child's school performance and results of developmental assessment.

**Developmental Assessment Instrument**	Mother's report^*^		
	**Reading**	**Writing**	**Arithmetic**
	**r_spearman_ (*P*-value)**	**r_spearman_ (*P*-value)**	**r_spearman_ (*P*-value)**
Kaufman Assessment Battery for Children (K-ABC; arithmetic)	n.a.	n.a.	0.29 (<0.0001)
Tavor Picture Naming Expressive Vocabulary Test	0.27 (<0.0002)	0.20 (<0.01)	n.a.
**Aleph-ad-Taf**			
Reading words—time	0.50 (<0.0001)	0.36 (<0.0001)	n.a.
Reading words—accuracy	0.32 (0.0002)	0.30 (0.0004)	n.a.
Reading paragraph—time	0.43 (<0.0001)	0.19 (0.030	n.a.
Reading paragraph—accuracy	0.36 (<0.0001)	0.20 (0.03)	n.a.
Writing, dictation—time	0.22 (0.01)	0.35 (<0.01)	n.a.
Writing, dictation—accuracy	0.24 (0.004)	0.39 (<0.0001)	n.a.

* *Skills in comparison to classroom peers (better, similar, or worse)*.

*n.a. indicates that the instrument was not intended to assess the specific skill*.

## Discussion

Constructing a large scale outcome study of IVF treatment poses the challenge of designing the most efficient evaluation of the children's development. The current report is intended to convey our experience planning such a study and to suggest a feasible research protocol for assessing development of children in the early school years. With the advances in perinatal technology, and in order to report accurate long-term outcomes of findings and interventions, many studies are faced with similar methodological challenges.

An apparently trivial issue in child development research is defining the typical or “normal” child. This term is usually assumed to define a reference group as compared to a group exposed to some treatment or intervention. Nevertheless, variation in child development is considerable and due to emerging diagnoses for conditions previously considered in the normal range, such as developmental coordination disorder (clumsy child) or learning disorder, it can be challenging to describe the limits between normal and atypical. Further, these limits often change. For example, with updated versions of the Diagnostic Statistical Manual (29) additional distinctions are made in diagnoses such as ASD and ADHD, thereby including increasing numbers of children in these categories. These distinctions are particularly critical in pediatrics, where variation within each modality, such as growth, behavior, and emotional development, can be broad without implying impairment. Attempts to achieve recognition of the “normal child” may lead to over-investigation and failure to reassure parents on the one hand, but risk missed diagnosis on the other hand (30). These issues are important in research as well as in the clinic.

It is a daunting task to attempt to gain a good picture of a child's development in the course of a half-hour telephone interview, or even in a 2-h examination. In addition, constructing a feasible research plan in terms of resources (manpower, time and budget) may become a task more difficult than making a diagnosis of a specific impairment. Nevertheless, the protocol presented here managed to cover the intended territory.

Regarding the telephone interview, almost all of those who consented were able to complete the interview within the allotted 30 min. Within this time frame the mothers reported on the children's health, educational framework, school performance and diagnosed disorders, and completed the four structured questionnaires describing motor development, emotional and behavioral problems and sensory integration functioning.

Two issues of concern arise with respect to this method: the validity of maternal report of current issues and the potential for recall bias regarding past health and development. Regarding maternal report, other studies have supported its value. Lai et al. (31) compared a telephone interview version of the HOME Observation Measurement of the Environment with an actual home visit. Of the 54 items common to both methods, agreement ranged from 73 to 100%. In a study validating the Colorado Learning Difficulties Questionnaire, Willcutt et al. (32) concluded that parent report showed strong convergent and discriminant ability on the Reading scale, with promising results for the Math scale and Social scales as well. In the current study, with respect to school achievement, comparisons with class peers were requested under the assumption that major disparities would be noticeable to the parents. This approach was also used by Wilson et al. in a follow-up study of young adults conceived by assisted reproductive technologies (33). Questionnaires employing parent report of children's behavioral problems have been widely used and validated (34–36).

With respect to maternal recall, several reports have confirmed its validity, albeit with some reservations. In a study of mothers of school-aged children born following IVF, Rice et al. (37) compared their recall of pre- and perinatal factors with medical records, and found a high degree of agreement (as per kappa statistics) regarding the majority of outcomes examined. Further, they found that maternal characteristics or children's behavior did not greatly influence the degree of agreement for most outcomes. Investigating reports of Pacific children's injuries up to six years postpartum, and matching them to New Zealand's national database, Robertson et al. (38) concluded that maternal recall reporting was found to be a valid measure, with no systematic under-reporting. They did, however, warn that there was some misinterpretation of questions, thus recommended vigilance in assessing similar questionnaires. Majewska et al. (39) conducted a prospective cohort study following children to three years of age, and concluded that maternal reports of age at achieving significant developmental milestones were sufficiently reliable to be used in clinical judgement. Nosaka et al. (40) found that mothers' reports of children's weight up to 10 years of age were accurate, with 94.9% within 10% of the measured weight. McCormick and Brooks-Gunn (41) considered the effect of children's current health status on maternal recall of infant events and concluded that although there were inaccuracies, they were not sufficient to invalidate the usefulness of the information acquired. To minimize the problem, in the present study information was requested in a chronological manner, and problems were defined by listing specific medical issues (e.g., respiratory, orthopedic, endocrine, etc.). Thus, it is recommended that this approach be considered in planning research based on maternal recall.

Regarding the in-person assessment, achieving compliance with examination of the child at the Child Development Center was an even greater challenge than obtaining agreement to the telephone interview. Only half of those who expressed willingness to be contacted to bring their child for the assessment did indeed make the appointment and arrived. No significant differences were found on most variables when comparing those who brought their child for examination with those who declined or did not arrive. Part of the reason for not actually arriving might have been due to limitations of the research team—limited days and hours at which the psychologists could receive children and the lack of a practical solution for conducting the examination in the family's home. These limitations were primarily due to financial constraints of the study budget. When recruiting the families for this phase of the research, promising to provide a letter summarizing the results of the evaluation proved to be a strong incentive to participate. In a study of mothers' willingness to enroll their infants in research studies, Maayan-Metzger et al. (42) also concluded that the benefit of learning about their children's development was a motivating factor for participating in studies that presented no perceived actual risk to the child.

The instruments selected for the psychologists' examination were feasibly completed within the 2-h time frame. This enabled assessment of the children's cognitive ability, executive functions, attention, and learning skills. The choice of instruments in such research is a major issue, and considerable deliberations and consultations were conducted before deciding on the final format. In light of the constraints noted above (particularly time limits and funding for skilled examiners) some instruments that would have been desirable had to be eliminated from the protocol. For example, inclusion of the Rey Verbal Auditory Learning Test (RVALT) (43) was considered, which could have added to the assessment of the children's executive and attention functions and didactic skills. However for this study it was considered too long for the time allotted for the examination. Assessing the children's motor skills, with an instrument such as the Peabody Developmental Motor Scales (44) could have examined that dimension more fully than did the DCDQ, however it would entail employing occupational or physical therapists and scheduling an additional session, which was not feasible under the circumstances.

The protocol for the parent interview included almost 200 items. On the one hand this could impact the response quality, but on the other hand it was considered important to cover as many areas of the child's development as possible. The plan was to limit the interview time to 30 min, and in actuality this limit was met. Further, of the 759 interviewees, only 20 (2.6%) requested to halt the interview before the end. If there is concern regarding “interviewee fatigue,” such that the answers might become routine or evasive, researchers might decide to place the less critical items toward the end of the interview, or alternatively to prepare two or three versions of the questionnaire with variations of the order of the items.

A potential limitation of this study is that it was conducted in Israel and in the Hebrew language. Thus it may not be possible to replicate this study in other countries without modification, and where possible validated translations of instruments should be employed. On the other hand, where the developers of the questionnaires and tests considered the scores to be age- or grade-dependent this was taken into account when scoring. These standards may vary across countries, cultures, and educational systems.

Although it would have been preferable to have Israeli norms for all instruments, they were not available for some of the instruments (e.g., K-BIT-2, ROCFT). However, this limitation was somewhat mitigated by the fact that they primarily included tasks with emphasis on visually-based processes rather than on language skills; thus it was expected that Israeli children would not perform significantly differently than those of other developed countries. Further, in the current research the focus was on comparing two groups (IVF and SC), thus comparison to national norms was less important.

Another compromise made was the use of the K-BIT-2 in place of a more comprehensive test of intelligence, such as the KABC or the Wechsler Intelligence Scale for Children (45), which have the advantage of Israeli norms. However this was done due to the time constraints noted and it was felt that the K-BIT-2 was a reasonable trade-off, especially considering that the focus of the research was to clarify any significant difference between the two study groups.

Since this study employed more than one psychologist for the examinations, there was some concern about reliability of scoring. For most of the instruments this was not an issue, since the criteria were clearly objective (correct or incorrect response). However with respect to the ROCFT and the VMI, for which subjective judgement is required and correctness is not clearly delineated, interrater reliability was considered. Those tests posed two problems: (1) that there would be errors of judgement, and (2) there would be differences in judgement between the examiners. In order to minimize these risks, the results were reviewed by a supervising psychologist to assure accuracy and consistency. With regard to the ROCFT, this test has two components—copying and organizing. In this study only the copying aspect was scored. It would have been desirable to assess the organization skills as well, however scoring is not clearly defined and questions arose regarding the various tasks; since uniformity of scoring was not achieved, this aspect was not included in the database or in the letter to the parents.

Although the allotted time for the examinations was 2 h, and most were completed within this period, the psychologists noted that some children became quite fatigued toward the end of the session, which might have impacted the scores on the last instruments presented. For this reason attempts were made to schedule the appointments early in the day, but it was not always possible due to the parents' or the psychologists' constraints. In addition, whereas the protocol called for presenting the Aleph-ad-Taf Hebrew reading and writing test at the end, the psychologists noted that this was a rather difficult test and perhaps something simpler, such as the VMI should have been presented last. However, the risk would remain, i.e., poorer results on this exam. It might be concluded that a 2-h session is too long for children in this age group, but then the issue of covering as many domains as possible remains unresolved. This is a cost-benefit equation that must be taken into account.

The dual aspect of this research—interview and examination—offered the opportunity to note the extent to which the examinations confirmed the maternal report, and consideration of the ability to depend on parent reports in studies where examinations are not feasible. An interesting and potentially important finding was noted when comparing the information provided in the telephone interview and the results of the examination. The mothers were asked to describe their children's school performance in the fields of reading, writing and arithmetic. Comparing the mothers' assessments to their children's performance upon examination showed a significant degree of agreement on some of the sub-tests. However on some of the examinations this was not found, and on others although the findings were significant, the correlation coefficients were quite low. It is possible that a more detailed inquiry, or finer resolution than the three options offered (better, same, or worse than peers) would improve the degree of agreement. The mothers' reports of learning disorder and ADHD diagnoses were confirmed by sections of the TEA-Ch examination, as were the children's height and weight measurements. Even in this age group, when part of children's abilities are expressed at school, most mothers in this study had a fair impression of their children's strengths and difficulties, as well as the need for further consultation; thus the phone interview responses were quite accurate. This finding lends support for a relatively low-budget study, relying on detailed phone interviews and considering that some elements of the examination may be redundant. For research purposes the telephone interview can serve as a screening tool and the actual psychological evaluation might be recommended for children whose parents express concerns, which could lead to further testing for mainly fine motor, cognitive and social abilities.

In view of this large cohort of more than 750 children with primarily typical development, we recommend a simplified but comprehensive questionnaire and examination protocols for determination of typical development and impairments for school-age children.

## Author contributions

Authorship credit was based on substantial contributions to the conception and manuscript design: AF, SG, GH-Y, LB, BS, RR-E, LL-G, and LG. Acquisition and analysis of data, or interpretation of data; drafting the article or revising it critically for important intellectual content; final approval of the version to be published; accountable for all aspects of the work in ensuring that questions related to the accuracy or integrity of any part of the work are appropriately investigated and resolved: AF, SG, GH-Y, SF, RR-E, LL-G, and LG.

### Conflict of interest statement

The authors declare that the research was conducted in the absence of any commercial or financial relationships that could be construed as a potential conflict of interest.
